# Cardiorespiratory fitness attenuates the association between fatness and cardiometabolic risk in Chinese children

**DOI:** 10.3389/fendo.2024.1361447

**Published:** 2024-05-15

**Authors:** Ping-Ping Zhang, You-Xin Wang, Jia-Yin Gu, Miao Xu, Ye Zhou, Hai-Jun Wang, Patrick W C. Lau, Hui Wang, Li Li

**Affiliations:** ^1^ Ningbo Center for Healthy Lifestyle Research, The First Affiliated Hospital of Ningbo University, Ningbo, Zhejiang, China; ^2^ Department of Maternal and Child Health, School of Public Health, Peking University, Beijing, China; ^3^ Department of Endocrinology and Metabolism, The First Affiliated Hospital of Ningbo University, Ningbo, Zhejiang, China; ^4^ Department of Sport, Physical Education & Health, Hong Kong Baptist University, Hong Kong, Hong Kong SAR, China; ^5^ Laboratory of Exercise Science and Health, Beijing Normal University-Hong Kong Baptist University United International College (UIC), Zhuhai, Guangzhou, China

**Keywords:** obesity, metabolism, fitness, children, mediator

## Abstract

**Background:**

Childhood obesity tends to persist into adulthood, predisposing individuals to cardiometabolic risk (CMR). This study aims to investigate the mediating role of cardiorespiratory fitness (CRF) in the associations between multiple fatness indicators and individual CMR markers and clustered CMR-score, and explore sex differences.

**Methods:**

We recruited 1,557 children (age: 8 to 10, male/female: 52.7%/47.3%) in September 2022 in Ningbo, China. Physical examinations, overnight fasting blood test, and CRF was evaluated. The CMR-score was calculated by summing age- and sex-specific z scores of four CMR markers, including mean arterial blood pressure, triglycerides, the total cholesterol to high-density lipoprotein cholesterol ratio, and homeostatic model assessment for insulin resistance. Generalized linear mixed models were used to identify the associations, mediation analyses were performed to dissect the function of CRF.

**Results:**

Partial correlation analyses revealed positive associations between high fatness indicators (including body mass index [BMI], BMI z score, body fat mass index [BFMI] and waist-to-height ratio [WHtR]) and increased CMR markers, whereas high CRF was associated with decreased CMR markers (all *P* < 0.05). In the mediation analyses, CRF emerged as a partial mediator, attenuating the relationship between four fatness indicators and CMR-score. Specifically, CRF mediated 6.5%, 7.7%, 5.3%, and 12.5% of the association between BMI, BMI z score, BFMI, WHtR and CMR-score (all *P* < 0.001). And the mediating effects of CRF between WHtR and four individual CMR markers was particularly robust, ranging from 10.4% to 21.1% (all *P* < 0.05). What’s more, CRF mediates the associations between WHtR and CMR-score more pronounced in girls than boys with a mediation effect size of 17.3% (*P* < 0.001).

**Conclusion:**

In Chinese children, CRF partially mitigates the adverse effects of fatness on CMR, underscoring the significance of enhancing CRF in children.

## 1 Introduction

The rising prevalence of obesity has emerged as a pressing public health concern (1, 2). Obesity that manifests in childhood tends to endure into adulthood, predisposing individuals to cardiovascular and metabolic risks (3), such as dyslipidemia, elevated glucose levels, and high blood pressure. Collectively, these factors contribute to over 30.0% of global mortality (4). Emerging evidence indicates that clusters of cardiometabolic risk (CMR) factors associated with obesity commence early in life, persisting from childhood into adulthood and potentially forecasting future occurrences of diabetes and CMR (5, 6). Investigating the factors associated with CMR during childhood is crucial for implementing proactive management and early-stage interventions.

Cardiorespiratory fitness (CRF), also known as cardiovascular fitness or aerobic fitness, is characterized by the maximum capacity of the cardiovascular and respiratory systems to deliver oxygen to the skeletal muscles during physical activity. As the importance of CRF is now well-established, it was identified as a fundamental indicator in the assessment of health in youth (7). It is widely accepted that higher CRF is beneficial for the prevention of cardiovascular and metabolic diseases in both children and adults (8, 9). Evidence in children demonstrated that even after adjusting for the impact of obesity, the protection effect of CRF on CMR persists (10).

To date, several studies conducted on children and adolescent have consistently demonstrated that fitness, in particularly CRF, is associated with lower CMR, even after accounting for the negative effects of excess body fat (11–15). Notably, boys generally have higher CRF than girls across all age groups (16), and ethnic disparities in CRF have also been highlighted (17). However, to our knowledge, few studies have delved into the sexual dimorphism in this context, and the majority of them have been conducted in Western countries, thus limiting their generalizability. While one study among Chinese children by Shang et.al (13) evaluated CRF through the 50m × 8 shuttle run test and explored its mediation effect, its comparability with other studies remains questionable, and the validity and reliability of this test have not gained widespread acceptance (7). In our study, the 20-m shuttle run test (20mSRT) will be employed, which is more widely used. Furthermore, there remains a gap in understanding the impact of various fatness exposures, how individual markers contribute to CMR as well as the clustered CMR-score, and it is imperative to explore these factors, particularly with regards to sexual differences.

Therefore, this study aims to explore the mediating role of CRF in the association between four fatness indicators and CMR-score with individual CMR markers in Chinese children, and explore sex differences.

## 2 Methods

### 2.1 Study design and participants

The current study conducted a cross-sectional analysis using baseline data from the “Optimizing Intervention Effects in Children and Adolescents in Ningbo” program. This program is a cluster randomized controlled trial designed to evaluate the effect of a comprehensive intervention on weight management in third-grade primary students (Registration No. at clinicaltrials.gov: NCT05482165). Participants were recruited from six primary schools in the Haishu, Yinzhou, and Zhenhai districts of Ningbo city. The baseline assessment was conducted prior to the allocation of schools to either the treatment or control group and included data from physical examinations and CRF tests. Children aged between 8 and 10 years were recruited in September 2022. All third-grade students were recruited to participant this lifestyle intervention program. However, participants meeting the following criteria were excluded: those with a medical history of heart disease, hypertension, diabetes, asthma, viral hepatitis, or nephritis; individuals with obesity caused by endocrine diseases or drugs; those with abnormal physical development or physical deformity; students unable to participate in school sport activities; and individuals who experienced weight loss due to vomiting or taking drugs during the past 3 months (18).

**Figure 1:**
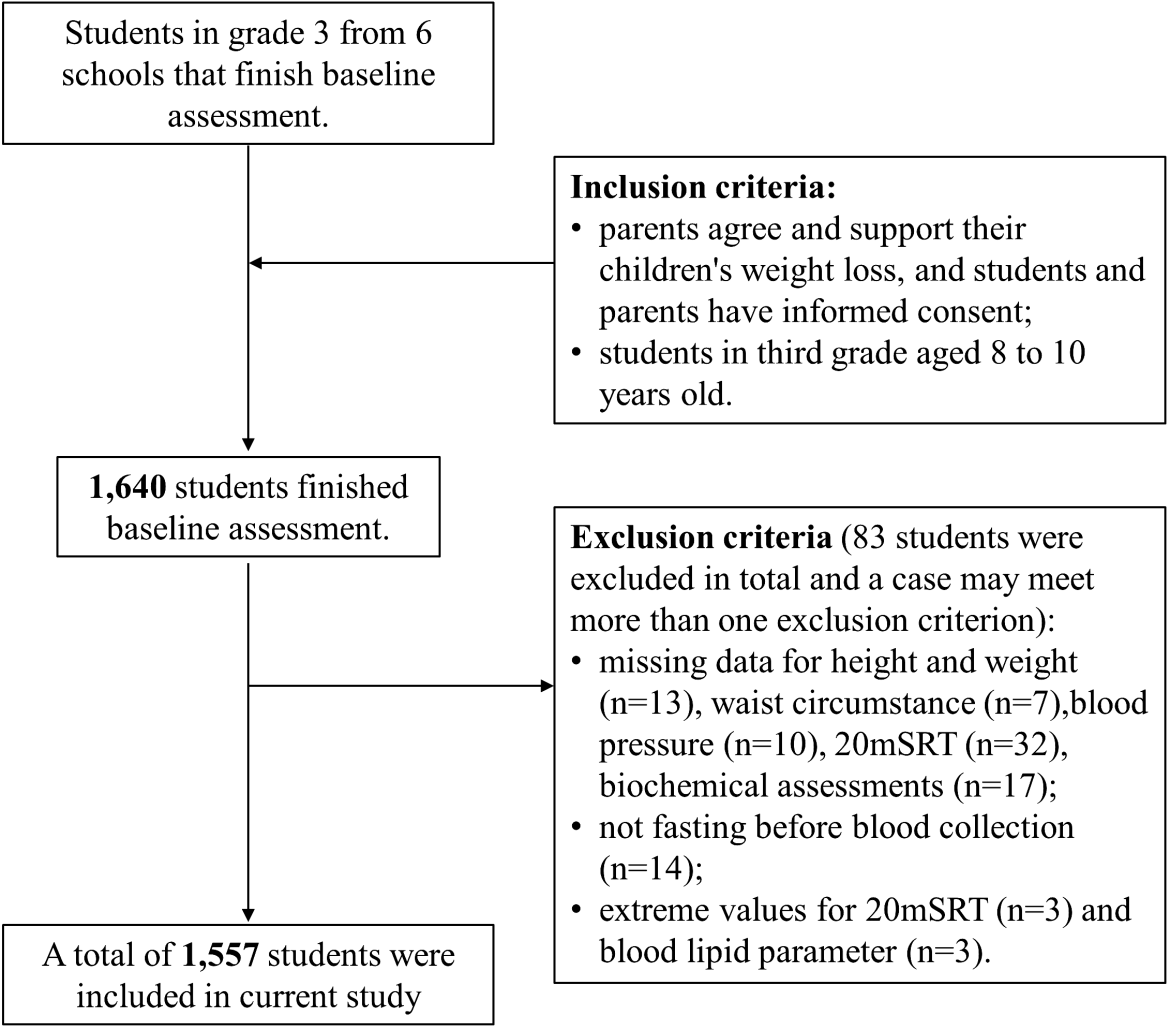
Flowchart for the selection of the study population.

The flowchart illustrating the selection of the study population in the current study is depicted in **Figure 1**. The program received approval from the Ethics Committee of the First Affiliated Hospital of Ningbo University (Approval No. 2021-R168). Written informed consent was obtained from all participating students and their primary guardians.

### 2.2 Measurements

#### 2.2.1 Anthropometry

Anthropometric measurements were conducted by trained staff from community healthcare centers following standard protocols at the participants’ schools. Diastolic blood pressure (DBP) and systolic blood pressure (SBP) were measured using an Omron digital sphygmomanometer (Omron HEM-7121, Kyoto, Japan) after the participants had rested for at least 5 minutes. Height was measured using a mechanical height meter with participants wearing no shoes. Waist circumference (WC) was measured as the circumference of the horizontal plane midway between the lowest rib and the iliac crest using a tape measure at the end of a normal expiration. Height and WC were all measured to the nearest 0.1cm. Weight and body fat mass were measured utilizing a bioimpedance analyzer (Inbody770, California, USA) with validity and reliability was demonstrated before (19, 20). The participants complied with the system commands by wearing only a single-layered shirt and shorts, standing barefooted on the electrode plates of the analyzer. They maintained a natural posture with both arms hanging down, holding the electrodes and placing their thumbs on them. Then the body composition test started and finished in 2 - 3 minutes.

Mean arterial blood pressure (MAP) was calculated as DBP + 1/3(SBP-DBP). The waist-to-height ratio (WHtR) was calculated as WC (cm) divided by height (cm). Body mass index (BMI) was calculated as weight (kg) divided by height (m) squared, and we calculated the age- and gender-specific BMI z score based on the WHO Child Growth Standards (21). Body fat mass index (BFMI) was calculated as body fat mass (kg) divided by height (m) squared. We selected BMI, BMI z score, BFMI and WHtR as the main fatness indicators in this study. BMI takes into account the relationship between height and weight, while BFMI considers body fat percentage, providing a more accurate reflection of obesity compared with BMI (22). We also used the z-score of BMI to eliminate the impact of age and gender, making BMI from different ages and genders comparable. WHtR is a good indicator of abdominal obesity and is closely related to cardiovascular disease risk (23). These indicators are complementary in assessing the impact of obesity on cardiovascular and metabolic risk.

#### 2.2.2 Biochemical assessments

Elbow vein blood samples (5mL) were obtained by trained nurses following a standard process during the morning of the physical examination at school. All blood parameters were collected after 10-14h of overnight fasting, and the collected blood was refrigerated at 4°C and transferred to examination on the same day. The blood was clotted for 20 - 30 min and centrifuged for10 - 15 min at 3,200 rpm, then the tests for different items were conducted with the remaining blood samples were divided and stored in -80°C refrigerator for further testing. Fasting insulin (FINS) levels were measured using a chemiluminescent method with an autoanalyzer (Roche Cobas E602 Immunology Analyzer, Basel, Switzerland). Fasting plasma glucose (FPG), triglycerides (TG), low-density lipoprotein cholesterol (LDL-C), high-density lipoprotein cholesterol (HDL-C), and total cholesterol (TC) were measured using an enzymatic method with another autoanalyzer (Beckman AU5800, California, USA). Homeostatic model assessment for insulin resistance (HOMA-IR) was calculated as [FINS (μU/L) * FPG (mg/dL)]/405. Additionally, the ratio of TC/HDL-C was calculated.

#### 2.2.3 Cardiometabolic risk score

Different methods were employed to calculate continuous CMR-score, and nearly all computed scores incorporated some measure of adiposity, lipids, metabolism, and/or blood pressure (24). In the current study, CMR-score were calculated by summing age- and sex-specific z scores of four individual markers contribute to CMR, including MAP, TG, TC/HDL-C ratio, and HOMA-IR (24). Adiposity-related indicators such as WC or BMI were not included in this score, due to the high correlation between WC and BMI in the mediation analysis for fatness indicators and CRF with CMR-score. The z scores for each index mentioned above were calculated as (value-mean)/SD separately for boys and girls and for each 1-year age group. Higher values of CMR-score indicated a greater cardiovascular and metabolic risk.

#### 2.2.4 Cardiorespiratory fitness

CRF was assessed using the 20mSRT, organized by trained professional project staff. This test has been widely used for assessing CRF in children and youth (25). In the 20mSRT, children ran between two lines 20 m apart, keeping pace with audio signals. The test comprises several stages (also called levels), each lasting approximately 1 minute, with each stage consisting of a number of 20-m laps (also called shuttles), and the laps was used to estimate CRF for each child. The speed started at 8.5 km/h and increased by 0.5 km/h every minute (1 min equals 1 stage) (26). The test ended either when a child failed to reach the end line concurrently with the audio signals on two consecutive occasions or when a child stopped due to fatigue. The overall number of laps completed was recorded.

### 2.3 Statistical analyses

Descriptive analyses were conducted for the characteristics of the participants and presented as means (SD). Student’s *t* test was employed to compare the differences between sexes concerning anthropometric measures, CRF, and cardiometabolic markers. Partial correlation coefficients (*r*), adjusted for age, sex, and school, were used as a pre-analysis to examine the associations between four fatness indicators (BMI, BMI z score, BFMI, and WHtR), CRF, and CMR markers.

**Figure 2:**
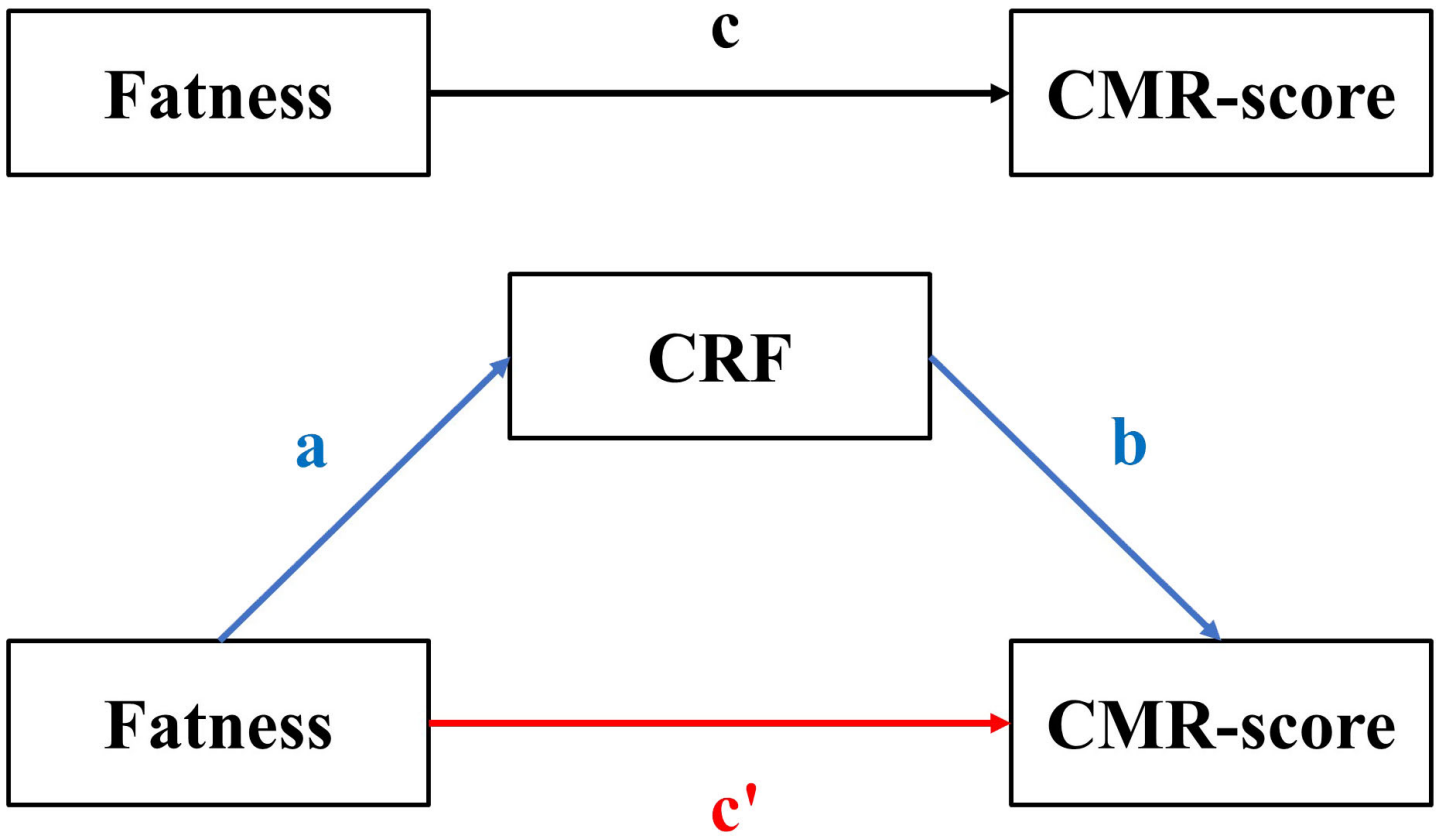
The mediation model pattern. The meaning of the lowecase letters were: c means the total effect, c’ means the direct effect, ab means the indirect effect.

To determine the associations between four fatness indicators, CRF, and CMR -score/-markers, a generalized linear mixed (GLM) model was utilized. This model included school-level random intercepts to account for the correlation due to the clustering of children within schools, as the survey was conducted in units of schools. All GLM models were adjusted for age and sex. The mediation model pattern is presented in **Figure 2**. The total effect (TE) of fatness indicators on CMR-score is represented by a black line, and the equation can be expressed as follows: equation (1) CMR-score ~ c × Fatness + covariables. The direct effect (DE) of fatness indicators on CMR-score is represented by the red line. The indirect effect (IE) of fatness indicators on CRF and CMR-score is represented by the blue line. The mediation equation included two parts: equation (2) CRF ~ a × Fatness + covariables and equation (3) CMR-score ~ c’ × Fatness + b × CRF. According to the theory of Baron et al. (27), a significant “indirect role” (mediation) was established when coefficients a, b, and c were significant, and then the mediation effects could be expressed as IE = a×b. The bootstrapping method (5000 samples) was used to examine whether the association between fatness indicators and CMR-score was mediated by CRF (28). The *mediation package* in R was used to estimate the DE, IE, and TE. The proportion of mediation was calculated as the ratio of IE to TE. A *P*-value < 0.05 for the two-sided test was considered statistically significant. All statistical analyses were performed using R 4.3.0 (R Core Team).

## 3 Results

### 3.1 Basic characteristics of the participants

We compared the characteristics of the 1,557 children included and 83 children excluded (**Supplementary Table S1** in **Supplementary Material**), and the excluded participants only reported to have a higher FPG compared to the included (*P* < 0.05). **Table 1** presents the basic characteristics of the participants. The children included in this study has an average age of 8.5 ± 0.3 years, 820 (52.7%) were boys. Notably, boys exhibited higher values in height, weight, WC, WHtR, BMI, body fat mass, BFMI, SBP, CRF, HDL-C, and FPG compared to girls (all *P* < 0.05). Conversely, girls demonstrated higher levels of TG and LDL-C (all *P* < 0.05). No significant differences in CMR-score were observed among genders (*P* = 0.789).

**Table 1:** Basic characteristics of the participants.

	Boys (n = 820)	Girls (n = 737)	Overall (n = 1557)	*P*
Age (year)	8.5 (0.3)	8.5 (0.3)	8.5 (0.3)	0.071
Height (cm)	133.2 (5.6)	132.1 (5.8)	132.7 (5.7)	**<0.001**
Weight (kg)	30.1 (6.5)	28.0 (5.6)	29.1 (6.2)	**<0.001**
Waist circumference (cm)	58.6 (7.3)	55.9 (6.1)	57.3 (6.9)	**<0.001**
WHtR	0.44 (0.05)	0.42 (0.05)	0.43 (0.05)	**<0.001**
BFP (%)	19.8 (8.4)	19.6 (7.2)	19.7 (7.9)	0.586
BMI (kg/m^2^)	16.9 (2.8)	16.0 (2.3)	16.5 (2.6)	**<0.001**
BMI group, n (%)				**<0.001**
Normal weight	585 (71.3)	605 (82.1)	1190 (76.4)	
Overweight	117 (14.3)	68 (9.2)	185 (11.9)	
Obese	118 (14.4)	64 (8.7)	182 (11.7)	
BMI z score	0.4 (1.4)	-0.1 (1.1)	0.1 (1.3)	**<0.001**
Body fat mass (kg)	6.4 (4.2)	5.8 (3.4)	6.1 (3.9)	**0.002**
BFMI (kg/m^2^)	3.6 (2.2)	3.3 (1.7)	3.4 (2.0)	**0.005**
SBP (mmHg)	102 (10)	101 (10)	101 (10)	**0.029**
DBP (mmHg)	63 (7)	63 (7)	63 (7)	0.629
MAP (mmHg)	76 (7)	75 (7)	75 (7)	0.162
CRF (laps)	28.2 (14.1)	25.2 (11.8)	26.8 (13.1)	**<0.001**
TG (mmol/L)	0.77 (0.34)	0.80 (0.32)	0.78 (0.33)	**0.030**
TC (mmol/L)	4.66 (0.81)	4.70 (0.87)	4.68 (0.84)	0.382
LDL-C (mmol/L)	2.79 (0.62)	2.86 (0.67)	2.82 (0.65)	**0.025**
HDL-C (mmol/L)	1.62 (0.29)	1.57 (0.29)	1.60 (0.29)	**<0.001**
FPG (mmol/L)	4.97 (0.37)	4.83 (0.34)	4.91 (0.36)	**<0.001**
FINs (pmol/L)	52.0 (28.6)	51.5 (30.1)	51.8 (29.3)	0.782
HOMA-IR	1.67 (0.97)	1.61 (1.01)	1.64 (0.99)	0.269

WHtR, waist-to-height ratio; BFP, body fat percentage; BMI, body mass index; BFMI, body fat mass index; CRF, cardiorespiratory fitness; SBP, systolic blood pressure; DBP, diastolic blood pressure; MAP, mean arterial blood pressure; TG, triglycerides; TC, total cholesterol; LDL-C, low-density lipoprotein cholesterol; HDL-C, high-density lipoprotein cholesterol; FPG, fasting plasma glucose; FINs, fasting insulin; HOMA-IR, homeostatic model assessment for insulin resistance.

Bold fonts indicate statistical significance.

### 3.2 Correlation between fatness indicators, CRF, and cardiometabolic markers

In the partial correlation analyses presented in **Table 2**, CRF was negatively associated with four fatness indicators (BMI, BMI z score, WHtR, BFMI) or individual CMR markers (MAP, TC/HDL-C, TG, HOMA-IR) after adjusting for age, sex, and school (all *P* < 0.05). Meanwhile, positive correlations were observed between multiple fatness indicators and CMR markers (all *P* < 0.05). The pre-analysis results of partial correlation coefficients fulfilled the prerequisites for mediating effect analyses. And the results of the between sexes analyses (**Supplementary Tables S2**, **S3**) were similar to the analyses of the entire population, the partial correlation of CRF and fatness indicator or CMR markers were significant (all *P* < 0.05) except for the association of MAP and CRF in boys.

**Table 2:** Partial correlation analyses among fatness indicators, CRF and cardiometabolic markers.

	CRF	BMI	BMI z score	WHtR	BFMI	MAP	TC/HDL-C	TG	HOMA-IR	
CRF	–	–	–	–	–	–	–	–	–	
BMI	-0.301*	–	–	–	–	–	–	–	–	
BMI z score	-0.283*	0.974*	–	–	–	–	–	–	–	
WHtR	-0.247*	0.714*	0.696*	–	–	–	–	–	–	
BFMI	-0.360*	0.954*	0.912*	0.704*	–	–	–	–	–	
MAP	-0.061*	0.191*	0.188*	0.101*	0.200*	–	–	–	–	
TC/HDL-C	-0.105*	0.241*	0.218*	0.180*	0.263*	0.068*	–	–	–	
TG	-0.165*	0.253*	0.227*	0.186*	0.256*	0.054*	0.414*	–	–	
HOMA-IR	-0.237*	0.483*	0.452*	0.316*	0.471*	0.107*	0.158*	0.299*	–	

CRF, cardiorespiratory fitness; BMI, body mass index; WHtR, waist to height ratio; BFMI, body fat mass index; MAP, mean arterial blood pressure; TC/HDL-C: total cholesterol to high-density lipoprotein cholesterol ratio; TG, triglycerides; HOMA-IR, homeostatic model assessment for insulin resistance.

* Indicates statistical significance.

– Indicate the variable’s correlation with itself or the repeated correlation coefficients are not shown.

### 3.3 Mediating effect of CRF on the association between fatness indicators and CMR

**Figure 3:**
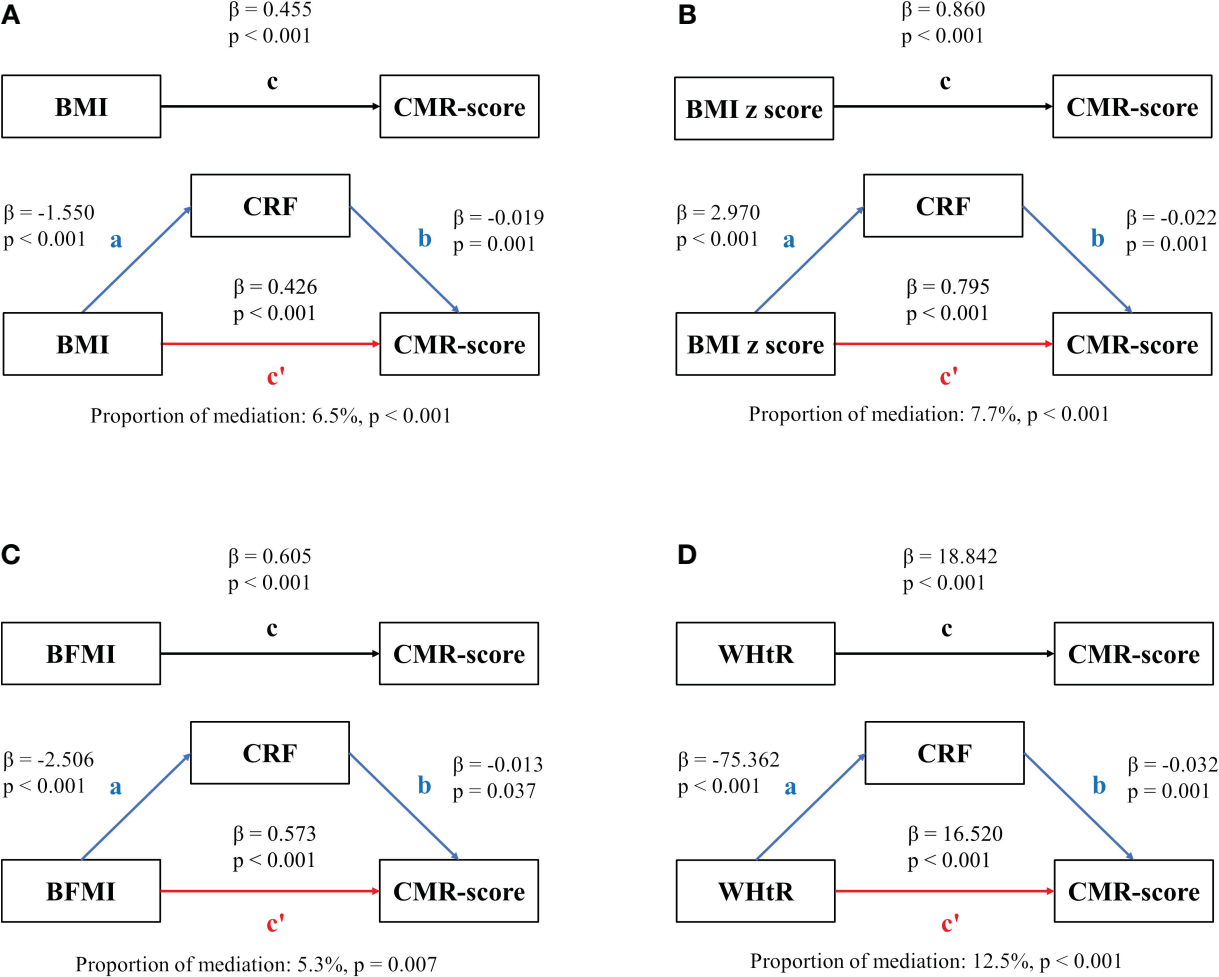
Mediation analysis. Contribution of CRF on the associations of fatness indicators and CMR-score. **(A)** The mediation between BMI and CMR-score; **(B)** The mediation between BMI z score and CMR-score; **(C)** The mediation between BFMI and CMR-score; **(D)** The mediation between WHtR and CMR-score. The meaning of the lowecase letters were: c means the total effect, c’ means the direct effect, ab means the indirect effect.

In general, all four fatness indicators were positively associated with CMR-score in our population, and the mediation analyses showed that CRF attenuated the association between fatness indicators and CMR-score (**Figure 3**). Of the four fatness indicators, CRF mediated 6.5% (95% CI: 3.3% to 9.6%, *P* < 0.001, **Figure 3A**), 7.7% (95% CI: 4.5% to 10.9%, *P* < 0.001, **Figure 3B**), 5.3% (95% CI: 1.5% to 9.0%, *P* = 0.007, **Figure 3C**) of the association between BMI, BMI z score, BFMI and CMR-score, and we found the largest mediating proportion of the association between WHtR and CMR-score (mediation effect: 12.5%, 95% CI: 8.4% to 16.6%, *P* < 0.001, **Figure 3D**). **Table 3** presents the mediation effect stratified by sex. The mediation effect for CRF between BFMI and CMR-score was only significant in girls with a proportion of 5.8% (95% CI: 0.9% to 10.6%, *P* = 0.021). Meanwhile, the proportion of mediation for CRF between WHtR and CMR-score was 9.5% (95% CI: 4.8% to 14.2%, *P* < 0.001) in boys and more pronounced in girls, with a proportion of 17.3% (95% CI: 9.6% to 25.0%, *P* < 0.001).

**Table 3:** Mediation effect of CRF on the associations between fatness indicators and CMR-score among sex.

Mediation effect		Boys			Girls		
		Estimate	95% CI	*P* value	Estimate	95% CI	*P* value
BMI	Indirect effect	0.026	(0.008, 0.044)	0.001	0.037	(0.015, 0.058)	0.001
	Direct effect	0.381	(0.327, 0.435)	<0.001	0.499	(0.423, 0.575)	<0.001
	Total effect	0.407	(0.353, 0.460)	<0.001	0.536	(0.464, 0.608)	<0.001
	Proportion of mediation%	6.3%	(1.9%, 10.6%)	**0.001**	6.7%	(2.7%, 10.7%)	**0.001**
BMI-z	Indirect effect	0.058	(0.023, 0.093)	<0.001	0.084	(0.041, 0.126)	<0.001
	Direct effect	0.709	(0.595, 0.822)	<0.001	0.947	(0.791, 1.103)	<0.001
	Total effect	0.766	(0.657, 0.876)	<0.001	1.031	(0.879, 1.182)	<0.001
	Proportion of mediation%	7.5%	(2.9%, 12.0%)	**<0.001**	8.0%	(3.9%, 12.1%)	**<0.001**
BFMI	Indirect effect	0.025	(-0.004, 0.054)	0.021	0.042	(0.007, 0.078)	0.021
	Direct effect	0.510	(0.437, 0.583)	<0.001	0.681	(0.585, 0.777)	<0.001
	Total effect	0.535	(0.469, 0.601)	<0.001	0.723	(0.631, 0.815)	<0.001
	Proportion of mediation%	4.7%	(-0.7%, 10.1%)	0.080	5.8%	(0.9%, 10.6%)	**0.021**
WHtR	Indirect effect	1.882	(0.941, 2.822)	<0.001	3.037	(1.699, 4.375)	<0.001
	Direct effect	17.742	(14.492, 20.993)	<0.001	14.364	(9.970, 18.758)	<0.001
	Total effect	19.624	(16.452, 22.796)	<0.001	17.401	(13.116, 21.685)	<0.001
	Proportion of mediation%	9.5%	(4.8%, 14.2%)	**<0.001**	17.3%	(9.6%, 25.0%)	**<0.001**

BMI, body mass index; BFMI, body fat mass index; WHtR, waist-to-height ratio.

Bold fonts indicate statistical significance.

Additionally, we explored the mediating effect of CRF between four fatness indicators and four individual CMR markers (detailed in **Tables 4**, **5**). We found that the mediating effect sizes of CRF between one of the fatness indicators “WHtR” and all four markers contribute to CMR were particularly robust, ranging from 10.4% to 21.1% (all *P* < 0.05). And in the further between sexes analyses, the mediation effect for the association between fatness indicators and MAP/TG were more remarkable in girls (**Supplementary Tables S4**, **S6**).

**Table 4:** Mediation effect of CRF on the associations between fatness indicators and MAP, TC/HDL-C.

Mediation effect		MAP			TC/HDL-C		
		Estimate	95% CI	*P* value	Estimate	95% CI	*P* value
BMI	Indirect effect	0.030	(-0.014, 0.075)	0.180	0.003	(0.001, 0.007)	0.057
	Direct effect	0.495	(0.352, 0.638)	<0.001	0.049	(0.038, 0.060)	<0.001
	Total effect	0.525	(0.390, 0.661)	<0.001	0.052	(0.042, 0.063)	<0.001
	Proportion of mediation%	5.6%	(-2.8%, 14.1%)	0.180	6.3%	(-0.2%, 12.8%)	0.057
BMI-z	Indirect effect	0.064	(-0.020, 0.149)	0.140	0.008	(0.001, 0.014)	0.021
	Direct effect	0.990	(0.700, 1.280)	<0.001	0.088	(0.067, 0.110)	<0.001
	Total effect	1.055	(0.782, 1.327)	<0.001	0.096	(0.075, 0.118)	<0.001
	Proportion of mediation%	6.0%	(-2.0%, 14.1%)	0.140	8.1%	(1.3%, 15.0%)	**0.021**
BFMI	Indirect effect	0.027	(-0.047, 0.100)	0.480	0.002	(-0.003, 0.008)	0.420
	Direct effect	0.661	(0.474, 0.848)	<0.001	0.071	(0.057, 0.086)	<0.001
	Total effect	0.687	(0.517, 0.857)	<0.001	0.074	(0.060, 0.087)	<0.001
	Proportion of mediation%	3.9%	(-7.2%, 15.0%)	0.480	3.2%	(-4.4%, 10.8%)	0.420
WHtR	Indirect effect	3.035	(0.878, 5.192)	0.006	0.251	(0.084, 0.418)	0.006
	Direct effect	11.291	(3.042, 19.540)	0.007	2.152	(1.534, 2.770)	<0.001
	Total effect	14.326	(6.367, 22.285)	0.001	2.403	(1.805, 3.001)	<0.001
	Proportion of mediation%	21.1%	(6.1%, 36.1%)	**0.007**	10.4%	(3.3%, 17.5%)	**0.006**

MAP, mean arterial blood pressure; TC: total cholesterol; HDL-C: high-density lipoprotein cholesterol; BMI, body mass index; BFMI, body fat mass index; WHtR, waist-to-height ratio.

Bold fonts indicate statistical significance.

**Table 5:** Mediation effect of CRF on the associations between fatness indicators and TG, HOMA-IR.

Mediation effect		TG			HOMA-IR		
		Estimate	95% CI	*P* value	Estimate	95% CI	*P* value
BMI	Indirect effect	0.003	(0.001, 0.005)	0.002	0.010	(0.005, 0.016)	<0.001
	Direct effect	0.030	(0.024, 0.037)	<0.001	0.177	(0.159, 0.195)	<0.001
	Total effect	0.033	(0.028, 0.039)	<0.001	0.187	(0.171, 0.204)	<0.001
	Proportion of mediation%	9.8%	(3.5%, 16.0%)	**0.002**	5.5%	(2.5%, 8.5%)	**<0.001**
BMI-z	Indirect effect	0.007	(0.003, 0.011)	<0.001	0.024	(0.013, 0.035)	<0.001
	Direct effect	0.054	(0.040, 0.067)	<0.001	0.332	(0.296, 0.368)	<0.001
	Total effect	0.061	(0.048, 0.074)	<0.001	0.356	(0.322, 0.390)	<0.001
	Proportion of mediation%	11.9%	(5.3%, 18.5%)	**<0.001**	6.8%	(3.8%, 9.8%)	**<0.001**
BFMI	Indirect effect	0.004	(0.001, 0.007)	0.031	0.010	(0.001, 0.018)	0.038
	Direct effect	0.042	(0.033, 0.050)	<0.001	0.232	(0.209, 0.255)	<0.001
	Total effect	0.046	(0.037, 0.054)	<0.001	0.242	(0.220, 0.263)	<0.001
	Proportion of mediation%	8.2%	(0.7%, 15.7%)	**0.031**	4.0%	(0.3%, 7.7%)	**0.038**
WHtR	Indirect effect	0.201	(0.101, 0.301)	<0.001	0.862	(0.556, 1.168)	<0.001
	Direct effect	1.458	(1.083, 1.833)	<0.001	6.727	(5.679, 7.775)	<0.001
	Total effect	1.659	(1.298, 2.020)	<0.001	7.589	(6.564, 8.614)	<0.001
	Proportion of mediation%	12.1%	(6.0%, 18.2%)	**<0.001**	11.3%	(7.3%, 15.3%)	**<0.001**

TG, triglycerides; HOMA-IR, homeostatic model assessment for insulin resistance; BMI, body mass index; BFMI, body fat mass index; WHtR, waist-to-height ratio.

Bold fonts indicate statistical significance.

## 4 Discussion

To the best of our knowledge, this study is the first to use the 20mSRT to assess CRF and illustrate the extent to which CRF may act as a mediator in the relationship between fatness indicators and CMR in Chinese children. This study revealed that CRF partially attenuate the associations between multiple fatness indicators and individual CMR markers and clustered CMR-score in Chinese children, with the mediation effect size for WHtR and CMR-markers/CMR-score being the largest. Meanwhile, the proportion of mediation were larger in girls, indicating that girls may benefit more from improvements in CRF.

Both obesity and CRF are crucial factors influencing cardiovascular health in childhood. We observed that higher fatness indicators were associated with a worse CMR level, while elevated CRF levels were linked to a better CMR level. These findings align with previous studies (29–32). Childhood obesity is a well-established marker for the clustering of metabolic risk factors, including high triglycerides, low HDL-C, high blood pressure, and dysglycemia (32). Although some therapeutic advances have emerged to address this global problem (33), longitudinal studies with comprehensive data, especially in children with obesity, are still essential to pinpoint the optimal timing for implementing mitigation strategies in children. These studies also suggest that defining a CRF threshold in children may help providers identify those at high risk of cardiometabolic disease, which could be considered in future study.

CRF has been recognized as a predictor of CMR and can be assessed through maximal or submaximal exercise tests. While maximal exercise tests, analyzed for gases, are considered the gold standard, they often require expensive equipment and well-trained staff, which may not always be readily available. In Chinese students aged 7 to 12 years old, the 50m × 8 shuttle run has traditionally been used to assess cardiorespiratory endurance. However, due to its test duration of less than 5 minutes, it may not accurately reflect the actual CRF level. The 20mSRT is currently the most widely used test in school settings worldwide to estimate CRF in children (26). In a review of 73 studies, it demonstrated moderate to high validity against the gold standard for CRF estimation (34). However, it should be concerned that evidence indicated performance in the 20mSRT has a shared variance with body size and fatness (8, 35), which may introduce confounding.

In our study, we observed that the mediation effect size was largest in the associations between one of the fatness indicators “WHtR” and CMR-score and CMR markers. Stoner et. al (14) also found the CRF as mediators in the fatness indicators (BMI, BFMI, and WHtR) and CMR-score in European adolescents (age: 14.1 ± 1.1 years old), the mediation effect sizes were all about 10.0%. Another study from European with children aged 8-11 years also concluded the similar mediation effect, with the fatness evaluated by BMI (15). Our study highlights the largest effect size for WHtR, possibly indicating that abdominal fat may be more important than total body fat, given that WHtR is frequently utilized as a proxy for central adiposity and has been validated as a significant predictor of morbidity (36). In comparison with BMI, WHtR demonstrates a stronger association with cardiovascular disease and provides a more accurate reflection of cardiovascular and metabolic risks (37).

Additionally, we noted that the mediation effect was more pronounced in girls than boys This suggests that interventions aimed at improving CRF may yield greater benefits in girls. In general, girls exhibit higher levels of abdominal fat than boys throughout childhood and adolescence (38). Consequently, improving CRF may potentially lead to easier reduction of abdominal fat in girls. Further research is warranted to investigate the biological mechanisms underlying this gender difference. Our analysis used BMI, BFMI, and WHtR as continuous variables rather than relying on specific clinical cut-offs, which allowed us to examine the dose-response relationships between fatness and CMR, as well as the mediating role of CRF. While our use of continuous variables provides valuable insights, future research could consider the use of clinical cut-offs.

The “fat-but-fit” hypothesis suggests that the risk of all-cause and cardiovascular disease mortality in individuals with obesity but who are fit (i.e., having CRF levels above the age-specific and sex-specific 20th percentile) is not significantly different from that of their normal-weight and fit counterparts (39). Musa et al. (40) found that both fatness and fitness (estimated through the 20mSRT) are independent predictors of blood pressure in Nigerian children. Of these two factors, fatness plays a more important role. Additionally, compared with fat-unfit children, participants with higher CRF had more favorable BP profiles. In our study, we observed that all four fatness indicators (BMI, BMI z score, BFMI and WHtR) partially attenuate the association between fatness and CMR-score in children, with the mediation effect of WHtR being the most substantial, accounting for 12.5%. WHtR is considered an accurate and simpler index for evaluating obesity in children and adolescents (41). To some extent, these findings lend support to the “fat-but-fit” hypothesis. Being fit plays a major role in cardiovascular health, and children benefit from both improved fitness and weight loss. Given that intervention during childhood potentially benefit most, it is critical to conduct relevant intervention studies in children as early as possible in future research.

Several limitations need to be considered of this study. Firstly, we did not estimate the pubertal status of the participants, however, all participant were from grade 3 in primary school with age of 8.5 years, for Chinese children, puberty starts at 9.65 years for boys and 10.65 years for girls (42). Secondly, the cross-sectional design inherently cannot eliminate bidirectional causality, and the narrow age range (8–10 years old) of the participants limit the generalizability, in that large and longitudinal studies may offer more conclusive evidence. Thirdly, there is a concern whether CRF measured by 20mSRT being confounded by body size and composition, but 20mSRT is applicable, convenient and was recommended in large-scale childhood population (43).

## 5 Conclusion

In summary, our study results support the promotion that children with excess fatness should pay attention to improving CRF to reduce the harm of fatness to the cardiometabolic system. The more pronounced mediation effect observed in girls suggests potential benefits of CRF interventions. For children and adolescents, addressing both short-term and long-term cardiovascular risks necessitates not only weight management but also the enhancement of cardiopulmonary fitness.

## Data availability statement

The raw data supporting the conclusions of this article will be made available by the authors, without undue reservation.

## Ethics statement

The studies involving humans were approved by Ethics Committee of the First Affiliated Hospital of Ningbo University. The studies were conducted in accordance with the local legislation and institutional requirements. Written informed consent for participation in this study was provided by the participants’ legal guardians/next of kin.

## Author contributions

P-PZ: Data curation, Formal analysis, Investigation, Software, Writing – original draft. Y-XW: Data curation, Formal analysis, Investigation, Methodology, Software, Validation, Writing – original draft. J-YG: Investigation, Methodology, Project administration, Resources, Writing – review & editing. MX: Data curation, Investigation, Supervision, Writing – review & editing. YZ: Data curation, Investigation, Supervision, Writing – review & editing. H-JW: Conceptualization, Resources, Writing – review & editing. PL: Conceptualization, Writing – review & editing. HW: Conceptualization, Data curation, Funding acquisition, Methodology, Resources, Writing – review & editing. LL: Conceptualization, Data curation, Funding acquisition, Investigation, Project administration, Resources, Supervision, Writing – review & editing.
