# Supplementary material for: Cardiorespiratory fitness attenuates the association between fatness and cardiometabolic risk in Chinese children
Source: Front Endocrinol (Lausanne). 2024 May 15;15:1361447. doi: 10.3389/fendo.2024.1361447 (PMC11133549; doi:10.3389/fendo.2024.1361447)
Supplement: Supplementary file 1 [file Table_1.docx]

**Table S1. Comparation of the baseline characteristic between children included (n=1557) and excluded (n=83) in analyses.**

|  | n | Included in analyses | n | Excluded in analyses | *P* |
| --- | --- | --- | --- | --- | --- |
| Sex, n (%) |  |  |  |  | 0.217 |
| Boys | 820 | 820 (52.7%) | 50 | 50 (60.2%) |  |
| Girls | 737 | 737 (47.3%) | 33 | 33 (39.8%) |  |
| Mean age (SD), year | 1557 | 8.5 (0.3) | 83 | 8.5 (0.3) | 0.943 |
| Mean height (SD), cm | 1557 | 132.1 (5.7) | 70 | 132.3 (6.0) | 0.918 |
| Mean weight (SD), kg | 1557 | 29.1 (6.2) | 70 | 30.4 (8.4) | 0.195 |
| Mean waist (SD), cm | 1557 | 57.3 (6.9) | 76 | 59.1 (8.9) | 0.055 |
| Mean BMI (SD), kg/m^2^ | 1557 | 16.5 (2.6) | 70 | 17.2 (3.6) | 0.114 |
| BMI group, n (%) |  |  |  |  | 0.122 |
| Normal weight | 1190 | 1190 (76.4%) | 46 | 46 (55.4%) |  |
| Overweight | 185 | 185 (11.9%) | 12 | 12 (22.3%) |  |
| Obese | 182 | 182 (11.7%) | 12 | 12 (22.3%) |  |
| Mean SBP (SD), (mmHg) | 1557 | 101 (10) | 73 | 102 (11) | 0.162 |
| Mean DBP (SD), (mmHg) | 1557 | 63 (8) | 73 | 66 (9) | 0.666 |
| Mean CRF (SD), laps | 1557 | 26.8 (13.1) | 51 | 31.7 (26.4) | 0.194 |
| Mean TG (SD), (mmol/L) | 1557 | 0.78 (0.33) | 66 | 0.89 (0.44) | 0.055 |
| Mean TC (SD), (mmol/L) | 1557 | 4.68 (0.84) | 66 | 5.02 (1.58) | 0.090 |
| Mean LDL-C (SD), (mmol/L) | 1557 | 2.82 (0.65) | 66 | 3.17 (1.47) | 0.063 |
| Mean HDL-C (SD), (mmol/L) | 1557 | 1.60 (0.29) | 66 | 1.57 (0.31) | 0.435 |
| Mean FPG (SD), (pmol/L) | 1557 | 4.91 (0.36) | 66 | 5.02 (0.43) | **0.040** |

*Abbreviations*: BMI, body mass index; SBP, systolic blood pressure; DBP, diastolic blood pressure; TG, triglycerides; TC, total cholesterol; LDL-C, low-density lipoprotein cholesterol; HDL-C, high-density lipoprotein cholesterol; FPG, fasting plasma glucose; 20mSRT: 20-m shuttle run test.

**Table S2. Partial correlation analyses among fatness indicators, CRF and cardiometabolic markers in boys.**

|  | CRF | BMI | BMI z score | WHtR | BFMI | MAP | TC/HDL-C | TG | HOMA-IR | |
| --- | --- | --- | --- | --- | --- | --- | --- | --- | --- | --- |
| CRF | - | - | - | - | - | - | - | - | - | |
| BMI | -0.310***** | - | - | - | - | - | - | - | - | |
| BMI z score | -0.293***** | 0.974***** | - | - | - | - | - | - | - | |
| WHtR | -0.249***** | 0.798***** | 0.778***** | - | - | - | - | - | - | |
| BFMI | -0.368***** | 0.961***** | 0.919***** | 0.781***** | - | - | - | - | - | |
| MAP | -0.037 | 0.162***** | 0.159***** | 0.114***** | 0.174***** | - | - | - | - | |
| TC/HDL-C | -0.120***** | 0.246***** | 0.225***** | 0.211***** | 0.267***** | 0.063 | - | - | - | |
| TG | -0.153***** | 0.256***** | 0.230***** | 0.245***** | 0.262***** | 0.024 | 0.426***** | - | - | |
| HOMA-IR | -0.235***** | 0.469***** | 0.451***** | 0.367***** | 0.456***** | 0.070***** | 0.153***** | 0.283***** | - |  |

*Abbreviations*: CRF, cardiorespiratory fitness; BMI, body mass index; WHtR, waist to height ratio; BFMI, body fat mass index; MAP, mean arterial blood pressure; TC/HDL-C: total cholesterol to high-density lipoprotein cholesterol ratio; TG, triglycerides; HOMA-IR, homeostatic model assessment for insulin resistance.

***** Indicates statistical significance.

**Table S3. Partial correlation analyses among fatness indicators, CRF and cardiometabolic markers in girls.**

|  | CRF | BMI | BMI z score | WHtR | BFMI | MAP | TC/HDL-C | TG | HOMA-IR | |
| --- | --- | --- | --- | --- | --- | --- | --- | --- | --- | --- |
| CRF | - | - | - | - | - | - | - | - | - | |
| BMI | -0.289***** | - | - | - | - | - | - | - | - | |
| BMI z score | -0.268***** | 0.975***** | - | - | - | - | - | - | - | |
| WHtR | -0.250***** | 0.635***** | 0.620***** | - | - | - | - | - | - | |
| BFMI | -0.349***** | 0.942***** | 0.899***** | 0.633***** | - | - | - | - | - | |
| MAP | -0.090***** | 0.232***** | 0.231***** | 0.107***** | 0.239***** | - | - | - | - | |
| TC/HDL-C | -0.094***** | 0.240***** | 0.216***** | 0.171***** | 0.266***** | 0.075***** | - | - | - | |
| TG | -0.180***** | 0.249***** | 0.224***** | 0.145***** | 0.248***** | 0.089***** | 0.407***** | - | - | |
| HOMA-IR | -0.241***** | 0.506***** | 0.460***** | 0.286***** | 0.498***** | 0.146***** | 0.164***** | 0.317***** | - |  |

*Abbreviations*: CRF, cardiorespiratory fitness; BMI, body mass index; WHtR, waist to height ratio; BFMI, body fat mass index; MAP, mean arterial blood pressure; TC/HDL-C: total cholesterol to high-density lipoprotein cholesterol ratio; TG, triglycerides; HOMA-IR, homeostatic model assessment for insulin resistance.

***** Indicates statistical significance.

**Table S4. Mediation effect of CRF on the association between fatness indicators** **and MAP between sexes.**

| Mediation effect | | Boys | | |  | Girls | | |
| --- | --- | --- | --- | --- | --- | --- | --- | --- |
|  |  | Estimate | 95% CI | *P* value |  | Estimate | 95% CI | *P* value |
| BMI | Indirect effect | 0.020 | (-0.040, 0.081) | 0.510 |  | 0.042 | (-0.023, 0.108) | 0.210 |
|  | Direct effect | 0.378 | (0.202, 0.554) | <0.001 |  | 0.688 | (0.465, 0.911) | <0.001 |
|  | Total effect | 0.398 | (0.228, 0.569) | <0.001 |  | 0.730 | (0.515, 0.945) | <0.001 |
|  | Proportion of mediation% | 5.2% | (-11.1%, 21.4%) | 0.510 |  | 5.8% | (-3.3%, 14.9%) | 0.210 |
| BMI-z | Indirect effect | 0.044 | (-0.068, 0.155) | 0.440 |  | 0.089 | (-0.038, 0.215) | 0.180 |
|  | Direct effect | 0.741 | (0.377, 1.104) | <0.001 |  | 1.430 | (0.960, 1.901) | <0.001 |
|  | Total effect | 0.785 | (0.441, 1.128) | <0.001 |  | 1.519 | (1.064, 1.974) | <0.001 |
|  | Proportion of mediation% | 5.4% | (-9.3%, 20.1%) | 0.440 |  | 5.7% | (-2.6%, 14.0%) | 0.180 |
| BFMI | Indirect effect | 0.012 | (-0.084, 0.109) | 0.810 |  | 0.044 | (-0.069, 0.158) | 0.450 |
|  | Direct effect | 0.521 | (0.278, 0.764) | <0.001 |  | 0.913 | (0.603, 1.223) | <0.001 |
|  | Total effect | 0.533 | (0.316, 0.751) | <0.001 |  | 0.957 | (0.671, 1.243) | <0.001 |
|  | Proportion of mediation% | 2.2% | (-17.1%, 21.5%) | 0.810 |  | 4.5% | (-7.3%, 16.2%) | 0.450 |
| WHtR | Indirect effect | 2.084 | (-0.849, 5.017) | 0.155 |  | 4.224 | (0.964, 7.483) | 0.012 |
|  | Direct effect | 11.492 | (1.006, 21.979) | 0.030 |  | 11.930 | (-1.028, 24.888) | 0.071 |
|  | Total effect | 13.576 | (3.462, 23.691) | 0.007 |  | 16.154 | (3.564, 28.744) | 0.010 |
|  | Proportion of mediation% | 15.2% | (-7.7%, 38.1%) | 0.160 |  | 26.2% | (4.8%, 47.6%) | **0.022** |

*Abbreviations*: BMI, body mass index; BFMI, body fat mass index; WHtR, waist-to-height ratio.

**Table S5. Mediation effect of CRF on the association between fatness indicators and TC/HDL-C between sexes.**

| Mediation effect | | Boys | | |  | Girls | | |
| --- | --- | --- | --- | --- | --- | --- | --- | --- |
|  |  | Estimate | 95% CI | *P* value |  | Estimate | 95% CI | *P* value |
| BMI | Indirect effect | 0.003 | (-0.001, 0.007) | 0.160 |  | 0.003 | (-0.002, 0.009) | 0.220 |
|  | Direct effect | 0.044 | (0.031, 0.057) | <0.001 |  | 0.058 | (0.039, 0.077) | <0.001 |
|  | Total effect | 0.047 | (0.035, 0.060) | <0.001 |  | 0.061 | (0.043, 0.080) | <0.001 |
|  | Proportion of mediation% | 6.4% | (-2.6%, 15.4%) | 0.160 |  | 5.5% | (-3.2%, 14.3%) | 0.220 |
| BMI-z | Indirect effect | 0.007 | (-0.001, 0.015) | 0.089 |  | 0.008 | (-0.002, 0.019) | 0.110 |
|  | Direct effect | 0.080 | (0.052, 0.107) | <0.001 |  | 0.106 | (0.067, 0.145) | <0.001 |
|  | Total effect | 0.087 | (0.061, 0.113) | <0.001 |  | 0.114 | (0.077, 0.152) | <0.001 |
|  | Proportion of mediation% | 8.2% | (-1.1%, 17.4%) | 0.089 |  | 7.4% | (-1.7%, 16.5%) | 0.110 |
| BFMI | Indirect effect | 0.002 | (-0.005, 0.009) | 0.500 |  | 0.002 | (-0.007, 0.011) | 0.630 |
|  | Direct effect | 0.064 | (0.046, 0.081) | <0.001 |  | 0.086 | (0.060, 0.112) | <0.001 |
|  | Total effect | 0.066 | (0.050, 0.082) | <0.001 |  | 0.088 | (0.065, 0.112) | <0.001 |
|  | Proportion of mediation% | 3.5% | (-7.3%, 14.2%) | 0.500 |  | 2.5% | (-8.3%, 13.2%) | 0.630 |
| WHtR | Indirect effect | 0.224 | (0.009, 0.440) | 0.042 |  | 0.281 | (0.018, 0.545) | 0.034 |
|  | Direct effect | 2.122 | (1.353, 2.891) | <0.001 |  | 2.200 | (1.123, 3.278) | <0.001 |
|  | Total effect | 2.347 | (1.597, 3.096) | <0.001 |  | 2.482 | (1.425, 3.538) | <0.001 |
|  | Proportion of mediation% | 9.5% | (0.4%, 18.6%) | **0.042** |  | 11.2% | (0.7%, 21.7%) | **0.034** |

*Abbreviations*: BMI, body mass index; BFMI, body fat mass index; WHtR, waist-to-height ratio.

**Table S6. Mediation effect of CRF on the association between fatness indicators and TG between sexes.**

| Mediation effect | | Boys | | |  | Girls | | |
| --- | --- | --- | --- | --- | --- | --- | --- | --- |
|  |  | Estimate | 95% CI | *P* value |  | Estimate | 95% CI | *P* value |
| BMI | Indirect effect | 0.002 | (-0.001, 0.005) | 0.170 |  | 0.004 | (0.002, 0.007) | 0.003 |
|  | Direct effect | 0.031 | (0.022, 0.039) | <0.001 |  | 0.030 | (0.020, 0.041) | <0.001 |
|  | Total effect | 0.033 | (0.025, 0.041) | <0.001 |  | 0.035 | (0.025, 0.045) | <0.001 |
|  | Proportion of mediation% | 5.9% | (-2.5%, 14.4%) | 0.170 |  | 12.7% | (4.3%, 21.2%) | **0.003** |
| BMI-z | Indirect effect | 0.005 | (0.001, 0.010) | 0.080 |  | 0.010 | (0.004, 0.015) | 0.001 |
|  | Direct effect | 0.054 | (0.037, 0.071) | <0.001 |  | 0.056 | (0.034, 0.077) | <0.001 |
|  | Total effect | 0.059 | (0.043, 0.075) | <0.001 |  | 0.065 | (0.044, 0.086) | <0.001 |
|  | Proportion of mediation% | 8.0% | (-0.7%, 16.8%) | 0.080 |  | 14.6% | (5.7%, 23.6%) | **0.001** |
| BFMI | Indirect effect | 0.001 | (-0.003, 0.006) | 0.550 |  | 0.006 | (0.002, 0.011) | 0.012 |
|  | Direct effect | 0.043 | (0.032, 0.054) | <0.001 |  | 0.041 | (0.027, 0.054) | <0.001 |
|  | Total effect | 0.044 | (0.034, 0.054) | <0.001 |  | 0.047 | (0.034, 0.060) | <0.001 |
|  | Proportion of mediation% | 3.0% | (-7.1%, 13.1%) | 0.550 |  | 13.4% | (3.2%, 23.6%) | **0.012** |
| WHtR | Indirect effect | 0.119 | (-0.013, 0.251) | 0.079 |  | 0.296 | (0.142, 0.450) | <0.001 |
|  | Direct effect | 1.824 | (1.343, 2.306) | <0.001 |  | 0.941 | (0.361, 1.521) | 0.002 |
|  | Total effect | 1.943 | (1.478, 2.408) | <0.001 |  | 1.237 | (0.665, 1.809) | <0.001 |
|  | Proportion of mediation% | 6.0% | (-0.7%, 12.7%) | 0.079 |  | 23.7% | (10.6%, 36.8%) | **<0.001** |

*Abbreviations*: BMI, body mass index; BFMI, body fat mass index; WHtR, waist-to-height ratio.

**Table S7. Mediation effect of CRF on the association between fatness indicators and HOMA-IR between sexes.**

| Mediation effect | | Boys | | |  | Girls | | |
| --- | --- | --- | --- | --- | --- | --- | --- | --- |
|  |  | Estimate | 95% CI | *P* value |  | Estimate | 95% CI | *P* value |
| BMI | Indirect effect | 0.010 | (0.002, 0.017) | 0.008 |  | 0.012 | (0.004, 0.020) | 0.005 |
|  | Direct effect | 0.157 | (0.135, 0.178) | <0.001 |  | 0.208 | (0.180, 0.236) | <0.001 |
|  | Total effect | 0.166 | (0.146, 0.187) | <0.001 |  | 0.220 | (0.193, 0.247) | <0.001 |
|  | Proportion of mediation% | 5.9% | (1.4%, 10.3%) | **0.008** |  | 5.4% | (1.7%, 9.1%) | **0.005** |
| BMI-z | Indirect effect | 0.022 | (0.008, 0.036) | <0.001 |  | 0.029 | (0.012, 0.046) | <0.001 |
|  | Direct effect | 0.298 | (0.253, 0.342) | <0.001 |  | 0.389 | (0.329, 0.449) | <0.001 |
|  | Total effect | 0.320 | (0.277, 0.363) | <0.001 |  | 0.418 | (0.359, 0.477) | <0.001 |
|  | Proportion of mediation% | 6.9% | (2.6%, 11.3%) | **<0.001** |  | 6.9% | (2.9%, 10.9%) | **<0.001** |
| BFMI | Indirect effect | 0.009 | (-0.003, 0.021) | 0.130 |  | 0.011 | (-0.003, 0.025) | 0.130 |
|  | Direct effect | 0.201 | (0.173, 0.230) | <0.001 |  | 0.281 | (0.243, 0.319) | <0.001 |
|  | Total effect | 0.211 | (0.184, 0.237) | <0.001 |  | 0.292 | (0.257, 0.327) | <0.001 |
|  | Proportion of mediation% | 4.4% | (-1.4%, 10.2%) | 0.130 |  | 3.8% | (-1.0%, 8.5%) | 0.130 |
| WHtR | Indirect effect | 0.756 | (0.368, 1.143) | <0.001 |  | 1.073 | (0.581, 1.566) | <0.001 |
|  | Direct effect | 6.936 | (5.627, 8.244) | <0.001 |  | 6.054 | (4.312, 7.795) | <0.001 |
|  | Total effect | 7.691 | (6.399, 8.983) | <0.001 |  | 7.127 | (5.395, 8.859) | <0.001 |
|  | Proportion of mediation% | 9.8% | (4.9%, 14.7%) | **<0.001** |  | 15.0% | (7.9%, 22.0%) | **<0.001** |

*Abbreviations*: BMI, body mass index; BFMI, body fat mass index; WHtR, waist-to-height ratio.
